# Production and Consumption of Hydrogen in Hot Spring Microbial Mats Dominated by a Filamentous Anoxygenic Photosynthetic Bacterium

**DOI:** 10.1264/jsme2.ME11348

**Published:** 2012-03-23

**Authors:** Hiroyo Otaki, R. Craig Everroad, Katsumi Matsuura, Shin Haruta

**Affiliations:** 1 Department of Biological Sciences, Tokyo Metropolitan University, Minami-Osawa 1–1, Hachioji-shi, Tokyo 192–0397, Japan

**Keywords:** microbial mats, hydrogen, electron cycling, ecosystem

## Abstract

Microbial mats containing the filamentous anoxygenic photosynthetic bacterium *Chloroflexus aggregans* develop at Nakabusa hot spring in Japan. Under anaerobic conditions in these mats, interspecies interaction between sulfate-reducing bacteria as sulfide producers and *C. aggregans* as a sulfide consumer has been proposed to constitute a sulfur cycle; however, the electron donor utilized for microbial sulfide production at Nakabusa remains to be identified. In order to determine this electron donor and its source, *ex situ* experimental incubation of mats was explored. In the presence of molybdate, which inhibits biological sulfate reduction, hydrogen gas was released from mat samples, indicating that this hydrogen is normally consumed as an electron donor by sulfate-reducing bacteria. Hydrogen production decreased under illumination, indicating that *C. aggregans* also functions as a hydrogen consumer. Small amounts of hydrogen may have also been consumed for sulfur reduction. Clone library analysis of 16S rRNA genes amplified from the mats indicated the existence of several species of hydrogen-producing fermentative bacteria. Among them, the most dominant fermenter, *Fervidobacterium* sp., was successfully isolated. This isolate produced hydrogen through the fermentation of organic carbon. Dispersion of microbial cells in the mats resulted in hydrogen production without the addition of molybdate, suggesting that simultaneous production and consumption of hydrogen in the mats requires dense packing of cells. We propose a cyclic electron flow within the microbial mats, *i.e.*, electron flow occurs through three elements: S (elemental sulfur, sulfide, sulfate), C (carbon dioxide, organic carbon) and H (di-hydrogen, protons).

Microbial mats are microbial ecosystems that often develop on submerged surfaces such as at the bottom of aquatic environments. Cells belonging to a range of species exist in close proximity to each other in microbial mats. Photosynthetic microbial mats are sometimes observed at hot springs where photosynthetically-driven energy transduction is often an important process. Organic compounds produced by phototrophs are utilized by other heterotrophic organisms. Clarification of the material and energy flows driven by phototrophic bacteria in hot spring microbial mats will provide a model for understanding how ecosystems develop and are sustained.

Nakabusa hot spring in Japan has been well-documented by geochemists and microbiologists in terms of its well-developed microbial mats (7–9, 11, 12, 19–22, 24, 30, 31, 40). The hot spring water is slightly alkaline (pH 8.3–8.9) and contains sulfide (~0.1 mM), sulfate (~0.1 mM) and low concentrations of organic compounds (0.4 mg L^−1^ total organic carbon) (24, 30, 31). Several types of microbial mats develop here under the hot spring water. At approximately 65°C, microbial mats found here lack cyanobacteria but contain the filamentous anoxygenic phototroph *Chloroflexus aggregans*(24). This bacterium is found worldwide in alkaline hot springs, including in Japan (7), Iceland (37), Italy (35) and North America (34).

Kubo *et al.*(24) reported that microbial mats found at 65°C in Nakabusa were dominated by *Chloroflexus* sp. and the aerobic sulfur- and hydrogen-oxidizing *Sulfurihydrogenibium* sp. *In situ* hybridization analysis of these mats (~3 mm in thickness) showed that the habitat of *Sulfurihydrogenibium* was limited to the surface of the mats but that *Chloroflexus* was distributed throughout; the biovolume of *Chloroflexus* was 34% at the surface and increased to 64% in the interior of the mat. This observation suggested that the mat surface was aerobic and that aerobic respiration by bacteria sufficiently depleted oxygen to allow the existence of anaerobic niches deeper within the mats. Several studies have identified sulfide production during anaerobic incubation of mat samples from Nakabusa indicating the existence of sulfate reducers (24, 30, 31). Sulfide has been shown to be utilized as an electron donor by *Chloroflexus* under anaerobic conditions with illumination (26, 27). Carbon dioxide stimulated this sulfide consumption suggesting photoautotrophic oxidation of sulfide by *C. aggregans*(24). On the basis of these results, Kubo *et al.*(24) proposed that an interspecies interaction between sulfide producers and sulfide consumers constituted a sulfur cycle within the mats. At present however, the compounds that may act as electron donors for sulfide producers within the mats remain unclear. Nakagawa and Fukui found that external hydrogen enhanced the sulfide production of mats collected from Nakabusa hot spring (30). It is expected that hydrogen is one of the candidates for the electron donor. In this study, we attempted to determine the electron flow within the mats in order to better understand the whole of energy flow in this microecosystem. A special focus of this study is interspecies hydrogen transfer, because hydrogen is an important electron donor in anaerobic ecosystems. We identified hydrogen producers and consumers in the mats using a combination of molecular ecological, microbiological, and biochemical approaches. Our results are integrated with previous findings on carbon and sulfur flows (24, 30, 31) and we propose here a complete cycle of electron flow within the microbial mats.

## Materials and Methods

### Study area and sampling

Nakabusa hot spring is located in Nagano prefecture in Japan (36°23′15N″, 137°45′00E″, 1,500 m elevation). Microbial mats develop on a flood control wall under hot spring water flowing out from cracks in the wall. The thickness of the mats that develop here at 65°C are approximately 3 mm. Mat samples were collected into glass bottles using sterilized spatulas and tweezers in August 2008. The glass bottles were filled with hot spring water. Samples were brought to the laboratory in the dark without cooling and processed within 6 h for analyses of biological activity and bacterial isolation. Pieces of the mats were stored at −20°C until use for DNA extraction. The pH of the hot spring water was 8.3, as determined with a pH sensor (AS-212, AsOne, Tokyo, Japan) just after collection at the sampling site.

### DNA extraction from microbial mats

Bulk DNA was isolated from the mats using a modified chloroform phenol extraction protocol as reported by Kubo *et al.*(24). Briefly, microbial cells were disrupted by freeze/thaw and bead-beating steps then further lysed using lysozyme and proteinase K. After bringing to 0.95 M NaCl and 1% (w/v) hexadecyl-trimethyl-ammonium bromide (CTAB), nucleic acids were extracted by successive chloroform-isoamyl alcohol and phenol-chloroform-isoamyl alcohol steps and precipitated with isopropanol (45). RNAs were removed with RNase A.

### Terminal-restriction fragment length polymorphism (T-RFLP) analysis for the domain *Bacteria*

Bacterial 16S rRNA genes were amplified from the total DNA of the mats using the *Bacteria*-specific primers EubB (41) and 907R (29). The primer, EubB was labeled with 6-carboxyfluorescein (FAM) at the 5′ end. PCR was performed using ExTaq (Takara, Otsu, Japan) with the following PCR program: 94°C for 3 min; 25 cycles of 94°C for 30 s, 52°C for 45 s, and 72°C for 1 min; and 72°C for 5 min. PCR products were digested with *Msp* I at 37°C for at least 16 h. An aliquot of the digested fragments was analyzed using an ABI 3130xl capillary DNA sequencer using the GeneScan mode (Applied Biosystems, Carlsbad, CA, USA) and Peak scanner software (Applied Biosystems). A peak height threshold of 50 fluorescence units was used in the analysis.

### Clone library analysis for the domain *Bacteria*

For molecular cloning of the bacterial 16S rRNA gene, PCR was performed with the *Bacteria*-specific primers EubB and 907R as described above with the exception that the final extension time for PCR was 10 min. After removing the primers, PCR products were cloned into the pTAC-1 vector (BioDynamics Laboratory, Tokyo, Japan) and transformed into *Escherichia coli* JM109 competent cells (Nippon Gene, Tokyo, Japan). To identify the distribution of unique clone sequences, the T-RFLP fragment length (T-RF) for each clone was determined as described above after direct amplification of cloned DNA from the *E. coli* colony. DNA sequences of clones belonging to each unique T-RF size were determined with the BigDye Terminator v3.1 Cycle Sequencing Kit (Applied Biosystems) on an ABI3130xl Genetic Analyzer (Applied Biosystems) after PCR amplification from *E. coli* colonies with primers T7 and RV. Homology searches for clones were performed using the BLAST program at the NCBI website (http://www.ncbi.nlm.nih.gov/blast/Blast.cgi).

### Clone library analysis for bacteria in the phylum *Thermodesulfo-bacteria*

PCR primers Therdeslfo_140F (5′-AAGGGTGGCTAATAC CGG-3′, *E. coli* positions 140–159) and Therdeslfo_863R (5′-AGCTTCGGCACAGAAAGT-3′, *E. coli* positions 863–846), were used to specifically detect 16S rRNA gene sequences of phylogenetic groups that have been detected from Nakabusa hot springs in the phylum *Thermodesulfobacteria*(24, 30), including the genera *Thermodesulfatator* and *Caldimicrobium* and uncultured bacterium clones related to *Thermodesulfobacterium* sp. OPB45 (16). These primer sequences were designed based on the alignment of available sequences in the phylum *Thermodesulfobacteria* and other taxa, *i.e.*, genera *Thermodesulfovibrio*, *Chloroflexus*, *Fervidobacterium*, *Thermotoga*, and *Sulfurihydrogenibium*. This primer set was evaluated using the web-based program Probe Match at The Ribosomal Database Project (http://rdp.cme.msu.edu/).

A second PCR using these primers was performed on the products of the PCR amplification of bacterial 16S rRNA genes from total DNA of the mats as described above. This PCR used similar conditions to the previous PCR with the exception that the annealing temperature was 57°C. A clone library was constructed and DNA sequences of clones were analyzed after PCR amplification from the *E. coli* colonies with vector primers T7 and RV as described above. For phylogenetic analysis, sequence alignments were made with ClustalW in MEGA5 (43). Neighbor-joining analysis with maximum composite likelihood was performed using MEGA5 and was bootstrapped 1,000 times.

### Hydrogen-producing activity of microbial mats

One gram (wet weight) of the microbial mat was placed into a 70-mL vial containing 10 mL sterilized artificial hot spring water. The dry weight of 1 g wet weight of the mat was 21±2 mg, determined gravimetrically after drying at 80°C overnight. The artificial hot spring water consisted of 1 mM NaCl, 1 mM Na_2_HPO_4_, 0.5 mM Na_2_SO_4_, 0.3 mM Na_2_S, and 1 mM NaHCO_3_ (pH 8.5). The vials were sealed with butyl rubber stoppers and aluminum seals after replacing the gas phase with N_2_ gas. Sodium molybdenum oxide, an inhibitor of sulfate reducer (10), or 2-bromo-ethane sulfonate (BES), an inhibitor of methanogens (6), was added to the vial (final concentration of 20 mM or 2 mM, respectively) when indicated. During incubation at 65°C under dark or light (incandescent lamp; 300 μmol photons m^−2^ s^−1^) conditions, a portion of the gas phase was periodically collected using a gas-tight syringe. The amount of hydrogen gas obtained was determined by gas chromatography (GC-14A TCD, Shimadzu, Kyoto, Japan; porapack Type Q 80–100, mesh 80–100, Waters, Tokyo, Japan). The analysis conditions were as follows; column temperature, 60°C; injection temperature, 80°C; detector temperature, 80°C; current, 80 mA; carrier gas, N_2_. To disperse microbial cells in the mats, vials with a magnetic stirrer bar were shaken vigorously by hand before incubation and the dispersion was checked by microscopy. During incubation, the dispersed mats were continuously stirred by a magnetic stirrer. A phase contrast microscope CX41 (Olympus, Tokyo, Japan) equipped with a digital camera (ARTCAM 130MI; Artray, Tokyo, Japan) was utilized to observe microbial cells of the mats.

### Isolation of fermentative bacteria

Mats were washed with sterilized phosphate-buffered saline (PBS; 8 g NaCl, 0.2 g KCl, 1.44 g Na_2_HPO_4_, 0.24 g KH_2_PO_4_ per liter, pH 7.4), and 0.2 g (wet weight) was aseptically homogenized with a plastic pestle. The samples were suspended in 1 mL sterilized water and mixed with an agar medium in Petri dishes. The isolation medium (1 L) consisted of 0.25 g fructose, 0.25 g xylose, 0.25 g arabinose, 0.25 g Na-pyruvate, 1.0 g yeast extract (Nihon Pharmaceutical, Tokyo, Japan), 0.5 g Na-thiosulfate, 0.5 g NH_4_Cl, 0.2 g MgCl-6H_2_O, 0.07 g CaCl_2_-H_2_O, 0.03 g FeSO_4_-7H_2_O, 0.04% resazurin, 0.03 g Na_2_S-9H_2_O, 0.30 g cysteine-HCl-H_2_O and 15 g agar (pH 7.0). The agar plates were incubated at 65°C in the dark under anaerobic conditions. Anaerobic conditions were achieved using an oxygen absorber (Ever-Fresh, Torishige sangyo, Oita, Japan). A pure culture was obtained after two rounds of single colony isolation.

### Physiological and phylogenetic analyses of isolated strains

Bacterial isolates were anaerobically cultivated in 20 mL isolation medium (sugars and pyruvate were replaced by glucose) without agar in 30-mL test tubes with the gas phase replaced with N_2_ gas. Hydrogen and carbon dioxide gas production was measured using the same protocol for the detection of hydrogen production as described above. Organic acids produced in the culture broth were quantified by HPLC (SCR-101H column, Shimadzu; L-6200 pump, Hitachi, Tokyo, Japan; L-4000 UV detector, Hitachi, at 210 nm) (25). For 16S rRNA gene analysis, genomic DNA of bacterial isolates was extracted as described by Noll *et al.*(33). In brief, bacterial cells were disrupted with bead-beating (Ø0.1 mm zilconia-silica beads, BioSpec Products, Bartlesville, OK, USA). Genomic DNA was purified by phenol extraction, chloroform-isoamyl alcohol extraction and ethanol precipitation. PCR amplification of the 16S rRNA gene was performed as described above. The DNA sequences were determined using EubB and 907R as sequencing primers as described above.

### Nucleotide sequence accession numbers

The nucleotide sequences reported in this study were deposited in the DDBJ/EMBL/GenBank database with the following accession numbers: The unique 16S rRNA sequence from the isolated strains (AB685428) and clone library sequences (AB685429–AB685447).

## Results

### Bacterial composition of the *Chloroflexus*-dominated mats

To confirm the bacterial composition of the mats used in this study, a clone library of 16S rRNA genes was constructed after PCR amplification using *Bacteria*-specific primers. The 42 clones obtained were divided into 11 taxonomic groups (Table 1). T-RFs (fragment length in bp)=69, 83, 95, 117, 265, and 301 were found both in the clone library and direct T-RFLP analysis of the mats. The sum of the peak heights for these T-RFs accounted for 86.2% of the total peak height of the mat sample. Three additional T-RFs (T-RFs=158, 179 and 448) found in the T-RFLP profile of the mats were not recovered in clone library analysis; the peak height for each of these T-RFs was 5.4, 4.0, and 4.4% of the sample total peak height, respectively. Clones similar to *C. aggregans* (CP001337, 98.6% similarity) and the aerobic sulfur-oxidizer *Sulfurihydrogenibium azorense* Az-Fu1 (NR025259, 97.6% similarity) were detected as major members (31% and 21% of the total number of clones, respectively). No phototroph other than *C. aggregans* was detected in the clone library. Everroad *et al.* detected cyanobacteria as a major component of the Nakabusa mats below 58°C (2). T-RF=490, corresponding to this cyanobacteria, was also not observed in the present T-RFLP analysis. The predominant members of the mat community identified here are similar to those reported previously in mats that developed at Nakabusa at 65°C (24). In addition to these bacteria, the present clone library analysis indicated the predominance of a fermentative heterotroph, *Fervidobacterium riparium*, in the mats (21% of the total number of clones). Other clones accounting for <5% of the population in the clone library included aerobic heterotrophs, *e.g.*, *Thermus* spp., *Meiothermus* sp., and fermentative bacteria, *e.g.*, *Fervidobacterium pennavorans*, *Thermanaerothrix daxensis*, *Dictyoglomus turgidum* and *Ignavibacterium album*.

**Fig. 1:**
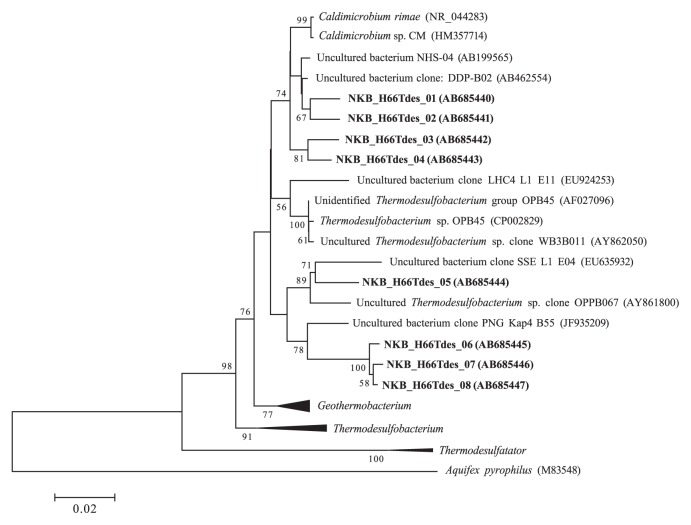
Neighbor-joining phylogenetic tree of the phylum *Thermodesulfobacteria* based on 16S rRNA gene sequences (*E. coli* positions 160–845). Clones from this study are in bold. Accession numbers are shown in parentheses. Bootstrap support values >50% are given. Scale bar shows 2% estimated sequence divergence.

Clone library analyses of the domain *Bacteria* did not detect sulfate- or sulfur-reducing bacteria from the phylum *Thermodesulfobacteria*, in contrast to previous reports on Nakabusa mats (24, 30). Specific primers were designed for PCR targeting the 16S rRNA genes related to phylogenetic groups that have been detected from Nakabusa hot springs in the phylum *Thermodesulfobacteria*. Clone library analysis of this targeted PCR revealed eight unique DNA sequences belonging to the *Thermodesulfobacteria* from the mats used in this study (Fig. 1). Four clones, NKB_H66_Tdes_05, 06, 07 and 08 were somewhat related to *Thermodesulfobacterium* sp. OPB45 (94.3–96.2% similarity), as reported by Kubo *et al.*(24). Recently, the genome sequence of *Thermodesulfo-bacterium* sp. OBP45 (CP002829) was suggested to have sulfate-reducing ability. The other clones NKB_H66_Tdes_ 01, 02, 03 and 04 were closely related to sequences previously detected by Nakagawa and Fukui (30). This latter group of clones likely is derived from the genus *Caldimicrobium* (97.2–97.7% similarity), which was recently reported to grow chemolithoautotrophically with hydrogen or organic acids in the presence of thiosulfate or sulfur (28).

### Hydrogen production from the mats

**Fig. 2:**
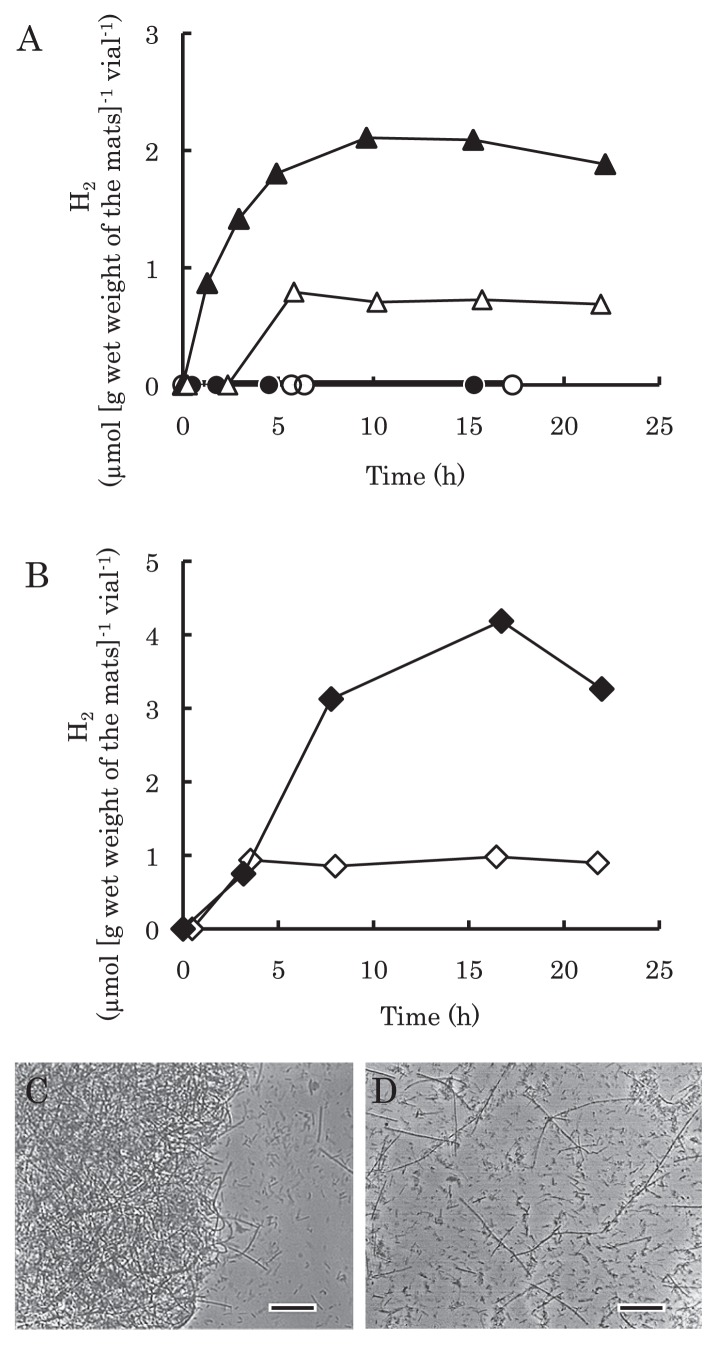
Hydrogen production by microbial mats in artificial hot spring water at 65°C. A, intact mats: ●, in the dark without molybdate; ○, in the light without molybdate; ▲, in the dark with molybdate; △, in the light with molybdate. B, dispersed mats: ◆, in the dark: ⋄, in the light. C, D, phase-contrast photomicrographs of mats. C, intact mats; D, dispersed mats. Scale bar = 20 μm.

The mats were incubated in artificial hot spring water at 65°C under anaerobic conditions. Mats that had not been physically disrupted did not produce hydrogen either in the dark or light (Fig. 2A); however, when molybdate (final 20 mM) was added, hydrogen was produced (Fig. 2A). Molybdate is an inhibitor of biological sulfate reduction (10). In the dark, the amount of hydrogen increased to 2.1 μmol (g wet weight of the mats)^−1^ vial^−1^ after 10 h incubation and remained relatively constant. Illumination suppressed the amount of the hydrogen production to less than 1 μmol (g wet weight of the mats)^−1^ vial^−1^ over the same time period. In contrast, the sole addition of BES, which inhibits methanogenesis (6), did not induce hydrogen production (data not shown).

Microscopic observation showed that the mats were dense with bacterial cells (Fig. 2C). The importance of the proximity of bacterial cells to each other for net hydrogen production was examined. Dispersion of the mats with a magnetic stirrer effectively disrupted the mat structure (Fig. 2D). Hydrogen production from the dispersed mats was observed even without the addition of molybdate under both light and dark conditions (Fig. 2B). The amount of hydrogen produced increased to 4.2 μmol (g wet weight of the mats)^−1^ vial^−1^ after 16 h incubation in the dark. This amount was larger than that from intact mats in the dark with molybdate. Suppression of this hydrogen production by illumination was observed for the dispersed cells (Fig. 2B), similar to observations for molybdate-treated mats.

Replication of the experiments in Fig. 2 was not possible due to the limited amount of mat material available from the sampling site; however, the effects of molybdate, BES, illumination and dispersion on hydrogen production, as shown in Fig. 2, were confirmed using microbial mats collected at the same site in July 2008 and August 2009. Comparative composition of bacteria in the mats was also assessed by T-RFLP (data not shown).

### Fermentative bacteria isolated from the mats

**Fig. 3:**
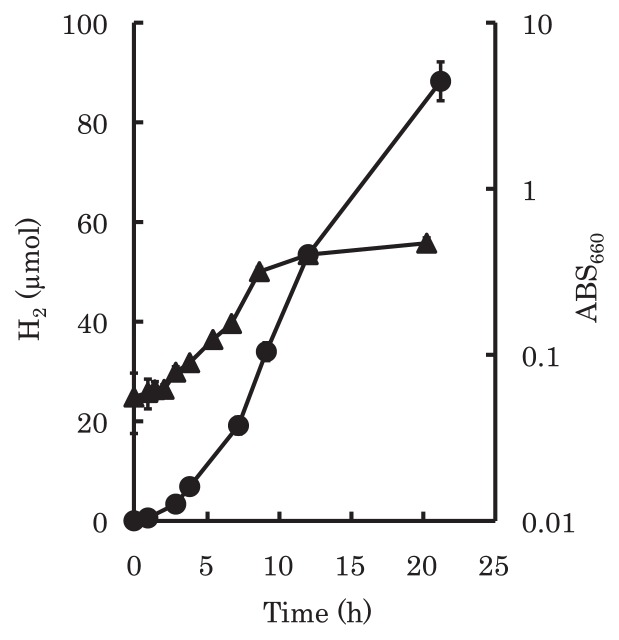
Growth and hydrogen production of isolated strain HO-65 under anaerobic conditions in the dark. ▲ ABS_660_; ● amount of hydrogen. Values are expressed as the means of three experiments. Error bars indicate SD.

Several isolates corresponding to clone NKB_H66_43 were isolated from the mats under fermentation conditions. The 16S rRNA gene sequences of these isolates were identical and shared 99.6% similarity with that of *Fervidobacterium riparium* 1445t^T^ isolated from a hot spring on Kunashiri Island (Kuril Islands, Russia) (36). A single representative, strain HO-65, was examined further. Hydrogen was produced during the growth of strain HO-65 on medium containing glucose (Fig. 3). Hydrogen production continued even after the culture entered the stationary phase from exponential growth. Lactate, acetate and carbon dioxide were also detected as fermentation products.

## Discussion

Predominance of *Chloroflexus* sp. in Nakabusa microbial mats at 65°C has been suggested by whole cell *in situ* hybridization and spectroscopic analyses (24, 40). Our clone library analysis showed that clones corresponding to *C. aggregans* accounted for 31% of the total number of clones (Table 1). Additionally, this analysis was the first to find that a fermentative bacterium, *Fervidobacterium riparium*, was a major component of a *Chloroflexus*-dominated mat. *Fervidobacterium* sp. strain HO-65 isolated from the mats produced hydrogen during anaerobic growth without illumination in media containing glucose. Hydrogen production from the mats was observed in the presence of molybdate or after cell dispersion (Fig. 2). Another possible pathway for hydrogen production in the mats is via nitrogen fixation (13). Photosynthetic nitrogen-fixing activity has been reported for microbial mats at Mushroom Hot Spring and Octopus Hot Spring in Yellowstone National Park, USA (38, 39); however, the suppression of hydrogen production by illumination suggested that photosynthetic nitrogen fixation did not occur in the mats used in this study. This indicates that *Fervidobacterium* works as a major hydrogen producer in this community. Additionally, *F. pennavorans* (clone NKB_H66_39) (3), *T. daxensis* (clone NKB_H66_24) (5), *D. turgidum* (clone NKB_H66_40) (42) and *I. album* (clone NKB_H66_03) (17) are possible candidates as hydrogen producers by means of their fermentative metabolism.

Based on clone library analysis, the population of *Fervidobacterium* was comparable to that of *C. aggregans* within the mats at Nakabusa. This fermenter seems to utilize organic matter efficiently derived from *C. aggregans. Chloroflexus aurantiacus* OK-70-fl, closely related to *C. aggregans*, has been reported to store polyglucose (15), and both C_5_ sugars (xylose, rhamnose) and C_6_ sugars (dominated by glucose, but also including arabinose) (44). These intracellular compounds might be provided through cell lysis by cell-lytic enzymes from other bacteria, *e.g.*, *Meiothermus* sp. (clone NKB_H66_34) (32). Although photoautotrophic growth of *C. aggregans* MD-66^T^ is yet to be reported, Kubo *et al.*(24) indicated that photoautotrophic sulfide oxidation occurs within mats dominated by *C. aggregans*. Genomic analysis of *C. aggregans* has provided evidence for the presence of a gene set required for the 3-hydroxypropionate autotrophic pathway (23). Thus, a possible cause of the suppression of net hydrogen production observed under illumination (Fig. 2) is hydrogen utilization by anoxygenic phototrophs, because *Chloroflexus* sp. has been reported to require either hydrogen or sulfide for its photoautotrophic growth (15, 26, 27).

*Ex situ* incubation of microbial mats collected previously from Nakabusa has suggested the activity of sulfate-reducing bacteria (24, 30, 31). The effect of molybdate on net hydrogen production (Fig. 2A) indicates that hydrogen produced in the mats was consumed by sulfate-reducing bacteria. Molybdate inhibits the first steps of sulfate reduction involving the activation of sulfate with ATP (10). In the present study, bacteria belonging to the phylum *Thermodesulfobacteria* were detected from the mats, as reported previously (24, 30). Bacterial members of the *Thermodesulfobacteria* likely work as sulfate reducers, utilizing hydrogen as an electron donor within the mats (Fig. 1). Of these, clones NKB_H66_Tdes_ 01, 02, 03 and 04 were related to the genus *Caldimicrobium*, which reduces thiosulfate or sulfur, but not sulfate, using hydrogen as an electron donor (28). This observation indicates that other hydrogen consumers, here a sulfur reducer, exist within the mats in addition to sulfate reducers. Supporting this, additional sulfide production was detected from the mats with the addition of sulfur globules under anaerobic and dark conditions (Otaki *et al.*, unpublished data). This sulfide production was stimulated by the external supply of hydrogen (Otaki *et al.*, unpublished data). In the mats, sulfur may be produced by the phototrophic oxidation of sulfide by *C. aggregans*. The genome sequence for *C. aggregans* (CP001337) shows that this bacterium has the gene encoding sulfide:quinone oxidoreductase (SQR) (4) but lacks the genes encoding dissimilatory sulfite reductase (DSR). Since DSR is essential for complete oxidation of sulfur compounds to sulfate (14), it appears that *C. aggregans* oxidizes sulfide to produce sulfur. Such production of sulfur by *C. aggregans* was supported by the microscopic observation of sulfur globules in *C. aggregans* cultures (data not shown).

The abundance and function of *Archaea*, including methanogens, in these mats has not been confirmed yet, because insufficient DNA fragments of archaeal 16S rRNA genes were recovered from the total DNA of the Nakabusa mats using PCR with *Archaea*-specific primers (2). In this study, no hydrogen production was observed in the presence of BES, indicating that the contribution of methanogens to hydrogen consumption within the mats was likely quite low in comparison with sulfate reducers. Although co-addition of BES and molybdate sometimes increased the amount of hydrogen produced compared with the sole addition of molybdate, this effect of BES was not observed in every mat sample tested. Further analyses are required to elucidate the distribution of methanogenic archaea in Nakabusa microbial mats.

The dispersion of cells in the mats may interfere with the intercellular interaction between fermenters and hydrogen consumers allowing hydrogen gas to escape to the gas phase. The high density of bacterial cells in the mats should support interspecies hydrogen transfer. Hydrogen transfer between cells has been reported to require close proximity, *e.g.*, less than 2 μm was required for syntrophic metabolism between a propionate-oxidizing syntroph and a methanogen (18). Suppression of hydrogen production by illumination was observed from the dispersed mats (Fig. 2B). *C. aggregans* cells in the mats seem to be responsible for this suppression through the photosynthetic consumption of hydrogen.

**Fig. 4:**
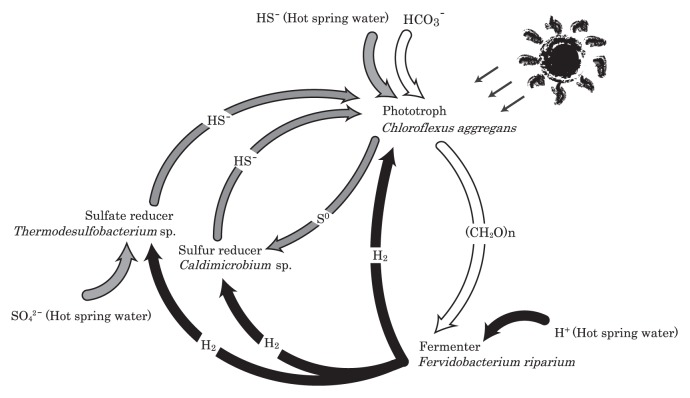
Model of material and electron flow in anaerobic regions within Nakabusa hot spring microbial mats. Carbon, hydrogen and sulfur flows are represented by white, black and gray arrows, respectively.

We propose a working model for material flow in *Chloroflexus*-dominated microbial mats in alkaline hot spring under anaerobic conditions (Fig. 4). During the day, *C. aggregans* appears to be the main primary producer, fixing inorganic carbon photoautotrophically. Organic compounds produced phototrophically are utilized by heterotrophs, *e.g.*, *Fervidobacterium riparium*. Fermentative metabolism then produces hydrogen, carbon dioxide and organic acids. The organic carbon products can further support heterotrophic growth in the mats, *e.g.*, *Chloroflexus* sp., while the hydrogen is simultaneously consumed by sulfate-reducing bacteria and *C. aggregans*. Hydrogen may also be utilized in part by sulfur reduction. Sulfate can be supplied from hot spring water. Sulfide produced by sulfate reduction is utilized by *C. aggregans* as it oxidizes sulfide to sulfur photoautotrophically. Sulfur is reduced to sulfide by sulfur-reducing bacteria. In aerobic areas at the surface layer of the mats, sulfide, sulfur and hydrogen can also be oxidized to sulfate or water by *Sulfurihydrogenibium* sp. (1, 24, 31).

These material flows are coupled to electron cycling within the microecosystem: 1) electrons are delivered from sulfide to organic compounds by the phototroph, 2) electrons from organic compounds are transferred to hydrogen by the fermenter, 3) electrons from hydrogen are transferred directly to the prototroph or through sulfide production to complete the electron cycle. This electron cycling connects three elemental cycles, *i.e.*, S, C and H, and in the anaerobic mats is driven by light and maintained by a continuous supply of all three elements from the source, with the biomass of the mats seemingly able to continue development as long as electron input from external sulfide is maintained.

## Figures and Tables

**Table 1:** Clone library of bacterial 16S rRNA gene

T-RF	Clone name	Accession no.	Closest cultured match	Similarity (%)	Phylum	Clone no.	%
69	NKB_H66_01	AB685439	*Chloroflexus aggregans* MD-66	98.6	*Choroflexi*	13	31
265	NKB_H66_43	AB685429	*Fervidobacterium riparium* 1445t	99.9	*Thermotoga*	9	21
95	NKB_H66_41	AB685430	*Sulfurihydrogenibium azorense* Az-Fu1	97.6	*Aquificiales*	9	21
512	NKB_H66_24	AB685431	*Thermanaerothrix daxensis* GNS-1	98.9	*Choroflexi*	2	5
117	NKB_H66_06	AB685432	*Thermus kawarayensis* KW11	98.5	*Deinococcus/Thermus*	2	5
484	NKB_H66_13	AB685433	Candidate division OP4	91.5	Unclassified	2	5
446	NKB_H66_34	AB685434	*Meiothermus* sp. L462	99.8	*Deinococcus/Thermus*	1	2
116	NKB_H66_32	AB685435	*Thermus* sp. Y55-10	99.6	*Deinococcus/Thermus*	1	2
263	NKB_H66_39	AB685436	*Fervidobacterium pennavorans* Ven5	98.6	*Thermotoga*	1	2
301	NKB_H66_40	AB685437	*Dictyoglomus turgidum* Z-1310	94.5	*Dictiyoglomi*	1	2
83	NKB_H66_03	AB685438	*Ignavibacterium album* Mat9-16	85.7	*Chlorobi*	1	2
						Total 42 clones	
